# Optimization of multi-AGV task allocation based on an improved PSO algorithm

**DOI:** 10.1371/journal.pone.0321616

**Published:** 2025-06-02

**Authors:** Yazhen Zhu, Qing Song, Meng Li

**Affiliations:** School of Electrical Engineering, University of Jinan, Jinan, China; Wenzhou University College of Mechanical and Electrical Engineering, CHINA

## Abstract

Research on task allocation for multiple automated guided vehicles (AGVs) in factory environments is a key topic in intelligent manufacturing. Existing studies often struggle to balance fairness and priority in task allocation, leading to low AGV utilization and high no-load distances. Moreover, the stability and applicability of task allocation algorithms in real-world production environments face significant challenges. To address these issues, a mathematical model is formulated with the objective of minimizing the no-load distances of all AGVs in material delivery tasks. The model is subsequently enhanced by incorporating task allocation balance and priority. To solve the optimization model, an improved particle swarm optimization algorithm is proposed, and extensive simulation experiments are conducted based on a real factory environment. By comparing the optimization results of the proposed algorithm with those of the latest multi-population genetic algorithm (MGA) and the market-based bundle task allocation method (MBTA), it is evident that both the proposed algorithm and MGA achieve higher AGV utilization and shorter total task completion times than MBTA, while also optimizing no-load distances. Although the running time of the proposed algorithm is slightly higher than that of MBTA, it is significantly lower than that of MGA, and its overall performance in reducing no-load distances and enhancing AGV utilization is superior to that of MGA. The proposed method can be applied to guide multiple AGVs in multi-material delivery tasks in real factory environments.

## Introduction

With the continued popularization of modern intelligent warehousing systems, efficient material transportation has become increasingly crucial. In this context, automated guided vehicles (AGVs) have emerged as a key tool for achieving efficient transportation of materials and goods, owing to their high flexibility and strong carrying capacity [1]. Utilizing AGVs to perform assigned tasks can enhance production efficiency, reduce transportation costs [2], and further elevate the level of intelligence in manufacturing.

In a real factory environment, a multitask allocation system for material transfer is an automated system that coordinates multiple tasks collaboratively. By considering priority tasks, adjusting the sequence of tasks allows multiple AGVs to complete tasks in less time, thereby improving overall transportation efficiency [3]. Given the presence of multiple AGVs, the multiple task allocation problem (MTAP) has garnered significant attention in intelligent manufacturing research due to its inherent complexity. In the MTAP problem, handling priority tasks, ensuring balanced allocation, and managing AGV loads are key challenges that significantly impact system efficiency. First, in a real factory environment, the timely processing of priority tasks plays a crucial role in the production process. However, traditional task allocation methods often overlook the importance of priority tasks, leading to delays in executing critical tasks. Second, unbalanced task allocation is a common bottleneck in multi-AGV systems. When different AGVs are assigned varying numbers of tasks, this results in reduced AGV utilization and decreased resource efficiency. Finally, AGV load management should focus on optimizing task sequences to minimize no-load time [4]. Therefore, the task allocation problem can be modeled as a multiconstraint optimization problem [5,6]. Faced with multiple transportation tasks and numerous AGVs, the AGVs will inevitably operate in a no-load state between tasks, which raises issues such as task execution sequencing and AGV load balancing—both of which directly impact the performance of the multi-AGV task allocation system [7].

In response to these challenges, scholars both domestically and internationally have conducted extensive research. Zhuang et al. [8] designed a task allocation system that combines offline and online methods. By using an improved genetic algorithm to determine the task set for each unmanned surface vehicle and employing a fast marching method to calculate the distance matrix between tasks, they reduced the fleet’s overall voyage, achieving a balanced task load distribution. Xu et al. [9] proposed an integrated solution framework based on graph theory to overcome the issue of serial solutions yielding only local optima. They modeled the path planning and task allocation problems as discrete graph models and employed a genetic algorithm to efficiently solve the mixed-integer programming problem. Additionally, they used a depth-first algorithm to detect loop states in the timing task graph, which helped assess the feasibility of the results. Internationally, Qamar et al. [10] introduced a task allocation method for multiple drones in SR scenarios, enhancing performance by incorporating compromise performance impacts with specific constraints. Javanmardi et al. [11] proposed a task allocation method, F-DTA, and used it as the fitness function for both the particle swarm and krill herd algorithms. They demonstrated the superiority of their method in execution time through simulations using the iFogSim2 simulator.

Among the various algorithms for solving task allocation problems, the particle swarm optimization (PSO) algorithm, an intelligent optimization method that simulates the foraging behavior of birds in nature, offers several advantages, including suitability for multiconstraint conditions, excellent parallel computing performance, robustness, and flexibility [12]. Despite these advantages, the traditional PSO algorithm has some drawbacks, such as a tendency to get trapped in local optima, the significant impact of initial particle distribution on search performance, and limited rationality in allocation results in real-world environments. To address these issues, many scholars have proposed various improvement strategies. Domestically, Wang et al. [13] proposed a strategy to escape local convergence based on a simulated annealing algorithm. Zhu et al. [14] optimized trajectory planning during the task allocation phase using a rapid search random number algorithm. Hua et al. [15] improved the PSO algorithm by combining it with the gradient descent method, enhancing both convergence accuracy and speed. Zhang et al. [16] proposed an adaptive genetic learning PSO algorithm, which generates high-quality elite particles through a genetic learning strategy, guiding the search to escape local optima. Internationally, Wei and Isa [17] proposed a PSO variant with dual-level task allocation, enhancing particle adaptability by assigning different search strategies to different dimensions. Mahdi et al. [18] introduced a multi-group hierarchical PSO algorithm based on Holonic organization, which strengthened the exploration and exploitation capabilities of PSO.

To further improve the rationality of allocation results and the convergence speed of the algorithm, this paper first designs an optimization based on task balance, building on the PSO algorithm. By introducing a penalty term, the optimization mitigates the issue of uneven resource allocation. Second, an optimized allocation strategy for priority tasks is proposed, where a distance matrix is constructed from the initial positions of the AGVs to the starting points of the priority tasks. Two different algorithms are then used in the front end of the improved PSO algorithm to assign priority tasks to AGVs, ensuring both the rationality and priority of task execution. Finally, a testing environment that more closely resembles a real factory setting is established to validate the proposed algorithm. The results indicate that, compared to the state-of-the-art multi-population genetic algorithm [19] and the market-based bundle task allocation method [20], the proposed algorithm effectively reduces no-load distances, improves task allocation balance, enhances the execution efficiency of priority tasks, and shortens total task completion times while maintaining a fast running time.

The rest of the paper is organized as follows. In the next section, we introduce the problem model for the material delivery scenario and establish a mathematical model for the task allocation system. We then present the basic process of the traditional PSO algorithm, followed by a detailed explanation of the proposed improvements to the PSO algorithm, focusing on allocation balance and task priority. The basic process of the improved algorithm is also provided. Next, we validate the effectiveness of the proposed algorithm in reducing no-load distances through simulation tests in typical scenarios, and assess its ability to handle allocation balance and task priority in multitask, multi-AGV environments. Finally, we summarize and conclude the study.

## Problem formulation

### Problem scenario model

In modern intelligent warehousing systems, AGVs are commonly used for material handling tasks between different locations within the factory. As the factory scales up and the number of tasks increases, the problem becomes more complex. In addition to ensuring the effective and efficient completion of tasks, it is also essential to improve the utilization rate of AGVs.

**Fig 1:**
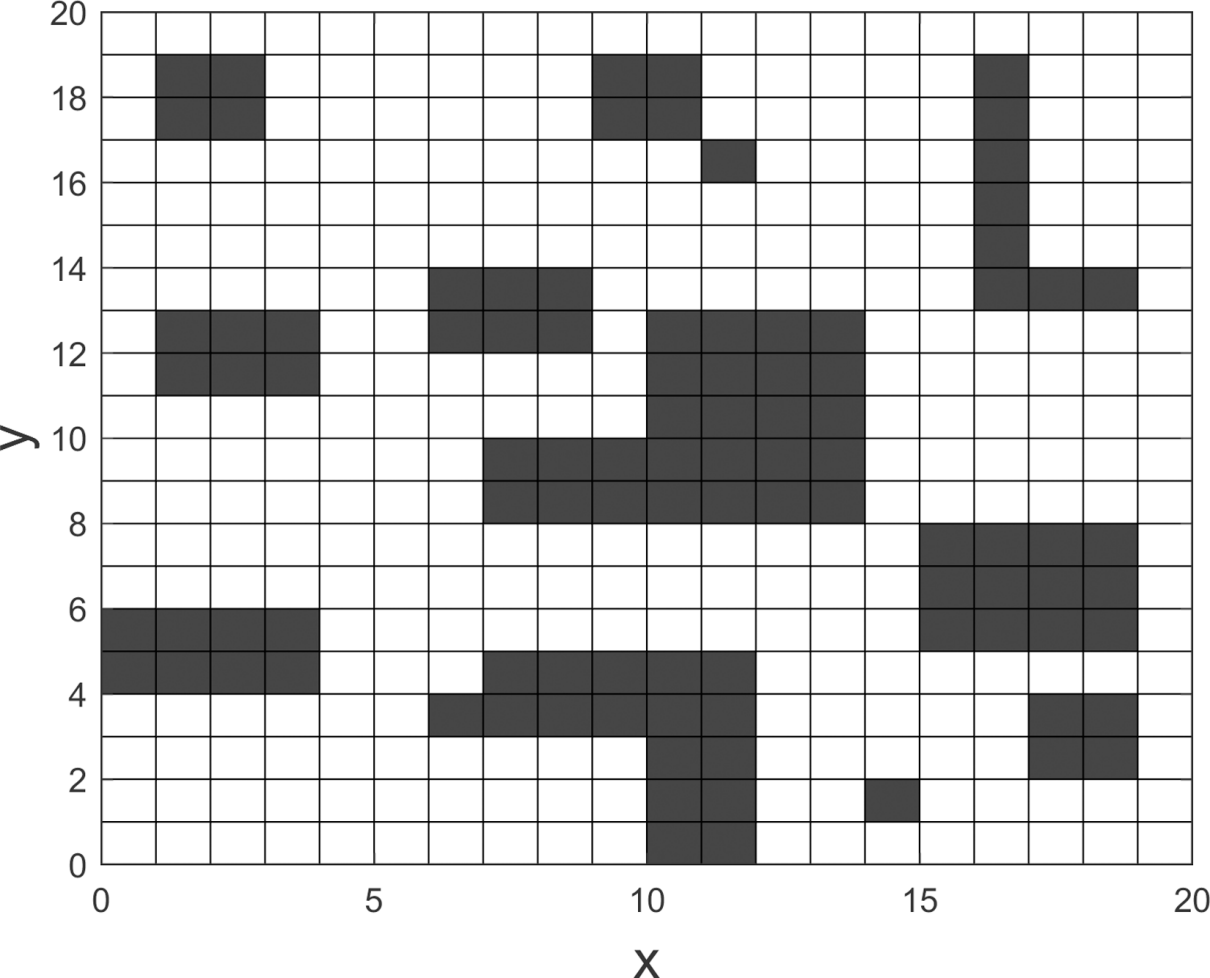
Grid map model of the factory area.

To quantify this problem, this paper first divides the factory area to establish a grid map model, as shown in Fig 1. Areas that are inaccessible to vehicles are represented by black grids in the figure.

This grid map model divides the factory area into 20 × 20 units, with a certain number of AGVs responsible for material handling tasks. In a multitask situation, an order pool stores all unexecuted tasks. In the order pool, tasks are arranged in sequences corresponding to the number of AGVs and assigned to the appropriate vehicles. Once a vehicle completes a task, it proceeds to the next task in the order pool. The vehicle then needs to calculate the optimal path between adjacent tasks, referred to as the no-load path between tasks. Clearly, a reasonable allocation of task sequences helps reduce the total length of AGV no-load paths and improves AGV utilization.

### Mathematical model

To optimize task sequences under the condition of multiple AGVs and tasks in a factory, an appropriate mathematical model needs to be established. The basic assumptions and constraints of the model are as follows:

- All AGVs have initial positions, which cannot serve as task starting or ending points. If no new tasks are assigned after a task is completed, the AGV will return to its initial position.
- Each AGV can only complete one task at a time.
- The same task can only be assigned to one AGV.
- Tasks vary in importance, with more important tasks given higher priority.
- All tasks must be assigned and executed.
- The loading and unloading times of the vehicles are not considered.
- All vehicles have the same driving speed.
- The turning time of the vehicles is not considered.
- The starting point of each task must be different from its endpoint.

Although some assumptions simplify the problem, they do not affect the correctness of the subsequent model or the solution algorithm. These assumptions are simply simplifications of certain calculations. Based on the problem scenario model established earlier, let v represent the driving speed of the AGVs, let r be the side length of each grid, and assume that the sides of the AGVs are of equal length, which is also equal to the side length of the grid. Let Z be the order pool set containing H material delivery tasks, with each task represented as $Z_{h}\in Z\;(h=1,2,...,H)$. The tasks that need to be prioritized are denoted as $\left\{Z_{1}^{*},\;Z_{2}^{*},\;\cdots ,\;Z_{f}^{*}\right\}$, where$Z_{h}^{*}\in Z\;(h=1,2,...,f)$, f is the number of priority tasks, and f<H. Each task has a starting point and an endpoint, represented by $S_{h}$ and $E_{h}$, respectively. For the starting and ending points of priority tasks, we add a superscript * for distinction, i.e., $S_{h}^{*}$ and $E_{h}^{*}$, respectively. Suppose there are a total of W AGVs, denoted by $AGV_{w}(w=1,2,...,W)$, each with an initial position $P_{w}$ and a sequence of tasks $\left\{Z_{1}^{w},\;Z_{2}^{w},\;\cdots ,\;Z_{k}^{w}\right\}$ to be executed sequentially, where $Z_{h}^{w}\in Z$ (h=1,2,...,k), and k is the number of tasks assigned to $AGV_{w}$. For the multi-AGV task allocation problem, k<H. Based on the preceding definitions, the segmented driving path of $AGV_{w}$ can be expanded as follows:


$$\mathrm{\backslash [}\Theta_{w}=\{(P_{w},S_{1}^{w}),(S_{1}^{w},E_{1}^{w}),(E_{1}^{w},S_{2}^{w}),...,(E_{k}^{w},P_{w})\}\mathrm{\backslash ]}$$ (1)

The pretask path of $AGV_{w}$ is defined as the path from its initial position $P_{w}$ to the starting point $S_{1}^{w}$ of the first task, with its length denoted as $L_{s}^{w}$. The posttask path of $AGV_{w}$ is the path from the endpoint $E_{k}^{w}$ of the last task back to its initial position $P_{w}$, with its length denoted as $L_{r}^{w}$. The no-load path between tasks for $AGV_{w}$ is the path between adjacent tasks, with its length denoted as $L_{b}^{w}$. Thus, we have:


$$\mathrm{\backslash [}L_{s}^{w}=L(P_{w},\;S_{1}^{w})\mathrm{\backslash ]}$$ (2)

$$\mathrm{\backslash [}L_{r}^{w}=L(E_{k}^{w},\;P_{w})\mathrm{\backslash ]}$$ (3)

$$\mathrm{\backslash [}L_{b}^{w}=\sum_{h=1}^{k-1}L(E_{h}^{w},\;S_{h+1}^{w})\mathrm{\backslash ]}$$ (4)

where the function L(x,y) computes the optimal path length from the starting point x to the endpoint y. For $AGV_{w}$, the total no-load distance, denoted as $L_{idle}^{w}$, is defined as the sum of the lengths of the pretask path, the no-load path between tasks, and the posttask path:


$$\mathrm{\backslash [}L_{idle}^{w}=L_{s}^{w}+L_{b}^{w}+L_{r}^{w}\mathrm{\backslash ]}$$ (5)

The time taken by $AGV_{w}$ to travel on the no-load path is called the no-load time, denote as $T_{idle}^{w}$. In comparison, the time taken by $AGV_{w}$ to transport goods is denoted as $T_{load}^{w}$. In a multi-AGV multitask scenario, if $AGV_{w}$ is not the last AGV to complete its tasks, there will be a waiting time until the last AGV finishes all its tasks, called the idle waiting time of $AGV_{w}$, denoted as $T_{wait}^{w}$. When all AGVs start executing tasks simultaneously, we have:


$$\mathrm{\backslash [}T_{wait}^{w}=\underset{w\in \{1,\cdots ,W\}}{\max}\;(T_{idle}^{w}+T_{load}^{w})-T_{idle}^{w}-T_{load}^{w}\mathrm{\backslash ]}$$ (6)

Excessive idle waiting time can lead to low utilization of individual AGVs, which will be further analyzed in the simulation test section.

To minimize AGV idle time, improve AGV utilization, and reduce the total task completion time, the basic objective of the multi-AGV multitask allocation and task sequence optimization system established in this paper is:


$$\mathrm{\backslash [}\min(Q)=\min(\sum_{w=1}^{W}L_{idle}^{w})\mathrm{\backslash ]}$$ (7)

The entire multi-AGV multitask task allocation and task sequence optimization system can be described as follows: calculate the task sequence $\left\{Z_{1}^{w},\;Z_{2}^{w},\;\cdots ,\;Z_{k}^{w}\right\}$ to be executed sequentially for each $AGV_{w}$ to minimize the total no-load distance, while considering priority tasks and adhering to the basic assumptions and constraints of the model.

## Solution methodology

Due to the complexity of the multi-AGV task allocation and task sequence optimization problem, particularly the need to balance task allocation and prioritize specific tasks, the PSO algorithm is well-suited for solving such challenges. Its parallel processing capabilities and strong adaptability make it an effective optimization method. Building on these strengths, this paper applies the PSO algorithm as the optimization approach and proposes improvements towards task balancing and task priority on the basis of the basic model.

### Traditional PSO algorithm

The PSO algorithm is inspired by the foraging behavior of bird flocks. It solves optimization problems by simulating group behavior in nature. Each individual, called a particle, moves through the search space and updates its position based on both individual and group experiences to find the optimal solution.

In the PSO algorithm, within an *H*-dimensional search space, assume there are *N* particles. The position and velocity of particle i(1≤i≤N) are each represented by an *H*-dimensional vector:


$$\mathrm{\backslash [}X_{i}=(x_{i1},x_{i2},...,x_{iH})\mathrm{\backslash ]}$$ (8)

$$\mathrm{\backslash [}V_{i}=(v_{i1},v_{i2},...,v_{iH})\mathrm{\backslash ]}$$ (9)

The best position found by particle *i* is denoted as $pbest_{i}$, and the best position found by the entire population is denoted as gbest.

For each particle *i* and each dimension h∈{1,2,⋯,H}, the velocity and position update equations are as follows:


$$\mathrm{\backslash [}v_{ih}(j+1)=\sigma v_{ih}(j)+c_{1}r_{1}(pbest_{ih}-xi_{h}(j))+c_{2}r_{2}(gbest_{h}-x_{ih}(j))\mathrm{\backslash ]}$$ (10)

$$\mathrm{\backslash [}x_{ih}(j+1)=x_{ih}(j)+v_{ih}(j+1)\mathrm{\backslash ]}$$ (11)

where $v_{ih}(j+1)$ is the velocity of particle *i* per dimension *h* at step j+1, and σ is the inertia weight, which reflects the particle’s ability to retain its previous velocity. A larger value of σ benefits global search, whereas a smaller value is advantageous for local search.$c_{1}$ and $c_{2}$ are learning factors that represent the influence of individual and group experience on the particle, respectively. $r_{1}$ and $r_{2}$ are random numbers in the range [0, 1] used to introduce randomness into the search. $c_{1}r_{1}(pbest_{ih}-xi_{h}(j))$ represents the influence of the individual’s known optimal solution, and $c_{2}r_{2}(gbest_{h}-x_{ih}(j))$ represents the influence of the group’s known optimal solution. $x_{ih}(j+1)$ is the position of particle *i* per dimension *h* at step j+1, which is equal to the position of the particle at step *j* plus the product of the velocity at step j+1 and the time step 1.

To prevent oscillation caused by excessive particle speed, the particle velocity is typically limited. During the iteration process, the fitness value of each particle is compared with its historical best value and the global best value at each step. If a particle’s fitness value is better, its historical best value and the global best value are updated. At the end of the algorithm’s iteration, the global best value and the corresponding position of the best particle are determined.

### Improved PSO algorithm

Research on improvements to the PSO algorithm primarily focuses on enhancing search capabilities, optimizing task allocation strategies, and improving adaptability to specific constraints. However, limited research has simultaneously addressed task allocation balance and priority. To fill this gap, this paper proposes a multi-AGV task allocation strategy based on an improved PSO algorithm. The strategy aims to address issues such as excessive no-load distance between tasks, low AGV utilization, unbalanced task distribution, and the need to prioritize specific tasks. The proposed method is designed to optimize task sequencing in multi-AGV, multi-task scenarios and achieve optimal AGV scheduling results.

In the improved PSO algorithm, the position of each particle represents a set of AGV task sequence allocations, which is an integer array of length *H*, where *H* is the total number of material delivery tasks. The *h*-th element in the array represents the AGV assigned to the *h*-th task, with values ranging from 1 to W, corresponding to $AGV_{1}$ through $AGV_{W}$. For example:


$$\mathrm{\backslash [}X_{1}=[1,3,4,5,2,3,5,1,4,2]\mathrm{\backslash ]}$$ (12)

Position *X*_1_ represents a scenario in which 5 AGVs execute 10 tasks. In this representation, the 1st task is assigned to the 1st AGV, the 2nd task is assigned to the 3rd AGV, and the remaining tasks follow a similar allocation pattern. The order of task allocation is crucial for multi-AGV scheduling. A well-organized order can optimize the paths of the AGVs, thereby reducing the total no-load distance and effectively addressing the problem of unbalanced task allocation. The speed of each particle represents the trend and magnitude of the change in the particle’s position, providing an update direction for each element in the position array. As shown in Formula (10), this speed value is influenced by parameters such as the individual optimal position, the global optimal position, the inertia weight, and the learning factor.

During particle initialization, each task is randomly assigned to an AGV by generating a random integer between 1 and W for each element in the array. This ensures comprehensive exploration of the solution space and prevents the algorithm from prematurely getting stuck in local optima. The particles’ velocities are also initialized randomly, allowing for a broad range of search directions.

For the multi-AGV task allocation and task sequence optimization problem, given the known initial positions of the AGVs, and with the objective function Q consisting of three components—the length of the pretask path, the length of the empty path between tasks, and the length of the posttask path—we can precalculate the following distance matrix to accelerate the algorithm:


$$\mathrm{\backslash [}M_{s}=\left[\begin{array}{cc} & L(P_{1},S_{1}),\;\;L(P_{1},S_{2}),\;...,\;L(P_{1},S_{H})\\ & L(P_{2},S_{1}),\;\;L(P_{2},S_{2}),\;...,\;L(P_{2},S_{H})\\ & \;\;\vdots \;\;\;\;\vdots \;\;\;\vdots \;\;\;\vdots \\ & L(P_{W},S_{1}),L(P_{W},S_{2}),...,L(P_{W},S_{H}) \end{array}\right]\mathrm{\backslash ]}$$ (13)

$$\mathrm{\backslash [}M_{b}=\left[\begin{array}{cc} & 0,\;L(E_{1},S_{2}),\;...,\;L(E_{1},S_{H})\\ & L(E_{2},S_{1}),\;0,\;...,\;L(E_{2},S_{H})\\ & \;\;\vdots \;\;\;\;\;\ddots \;\;\vdots \\ & L(E_{H},S_{1}),L(E_{H},S_{2}),...,0 \end{array}\right]\mathrm{\backslash ]}$$ (14)

where $M_{s}$ is the distance matrix from each AGV’s initial position to each task’s starting point. The element in the w-th row and h-th column represents the path length from the initial position of $AGV_{w}$ to the starting point of task h. $M_{b}$ is the distance matrix between tasks, with the main diagonal elements being 0. The values in the distance matrix are calculated using the A* algorithm, a heuristic search algorithm commonly used to find the shortest path and its length.

The speed is then updated according to the speed update formula, and the updated speed is applied to the current allocation strategy to obtain a new allocation. To ensure that each task is effectively assigned to an AGV, this paper applies value range constraints in the updated strategy, ensuring that the number of AGVs assigned to each task remains within the valid range of 1 to W. By repeatedly performing this process, the initial task sequence is rearranged based on the optimization objective function Q, ultimately resulting in the task allocation strategy with the minimum objective value.

#### Improvement on balanced task allocation.

Although minimizing the objective function Q can effectively reduce the length of no-load paths between AGVs, it may lead to some AGVs being overloaded with tasks, while others remain idle due to a task shortage. This imbalance results in resource waste, reduced system throughput, and prolonged overall task completion time. Therefore, in a multi-AGV system, it is crucial to effectively balance AGV loads in order to improve utilization. Based on Equation (7), we apply the following weighted transformation to the optimization objective function:


$$\mathrm{\backslash [}Q_{1}=Q+\lambda E\mathrm{\backslash ]}$$ (15)

$$\mathrm{\backslash [}E=\sum_{j=1}^{J}\left|k_{j}-\frac{H}{W}\right|\mathrm{\backslash ]}$$ (16)

where $Q_{1}$ is the optimization objective function with an added penalty term to mitigate imbalances. $k_{j}$ represents the number of tasks allocated to a certain AGV in thej-th iteration. By calculating and summing the deviation between $k_{j}$ and the average number of tasks, we obtain the total deviation E. Introducing a penalty coefficient λ for the total deviation allows us to balance the total no-load distance and task allocation fairness.

During the algorithm’s execution, the objective function uses a weighted sum of no-load distance and task allocation deviation as the optimization goal, ensuring that each iteration comprehensively considers both no-load distance and task allocation balance. This setup aims to mitigate the negative effects of unbalanced allocation by penalizing solutions with significant task allocation imbalances, thus promoting a more balanced task distribution. While minimizing the total no-load distance, the algorithm also balances the workload of each AGV, ensuring the efficient operation of the entire system.

#### Improvement on priority task allocation.

In modern intelligent warehousing systems, some tasks, although not at the forefront of the task sequence, need to be prioritized due to their importance. To ensure they are preferentially allocated to the most suitable AGV, we segment the optimization process of the PSO algorithm. Specifically, we add a greedy algorithm (or Hungarian algorithm) to the front end of the original PSO algorithm to handle the priority allocation of tasks. When the number of priority tasks f≤W, we use the greedy algorithm (or Hungarian algorithm) to assign priority tasks to each AGV in such a way that the total length of the AGV’s pretask paths is minimized. Then, the starting point of the AGV is updated to the endpoint of the priority task, and the PSO algorithm introduced in the previous section is used for further optimization. When the number of priority tasks f>W, a similar segmented optimization process can be applied.

To speed up the execution of the algorithm, we pre-construct the distance matrix $M_{f}$ from each AGV’s initial position to each priority task’s starting point, which quantifies the proximity of each AGV to the starting point of the priority tasks.


$$\mathrm{\backslash [}M_{f}=\left[\begin{array}{cc} & L(P_{1},S_{1}^{*}),\;\;L(P_{1},S_{2}^{*}),\;...,\;L(P_{1},S_{f}^{*})\\ & L(P_{2},S_{1}^{*}),\;\;L(P_{2},S_{2}^{*}),\;...,\;L(P_{2},S_{f}^{*})\\ & \;\;\vdots \;\;\;\;\vdots \;\;\;\vdots \;\;\;\vdots \\ & L(P_{W},S_{1}^{*}),L(P_{W},S_{2}^{*}),...,L(P_{W},S_{f}^{*}) \end{array}\right]\mathrm{\backslash ]}$$ (17)

The two front-end algorithms for assigning priority tasks to AGVs have different optimization effects. The greedy algorithm assigns each priority task to the nearest AGV, making it computationally efficient and fast. Due to the lack of a global perspective, the locally optimal solution obtained in the task allocation problem may not be globally optimal, potentially leading to some AGVs being assigned distant tasks and causing resource allocation imbalances. The Hungarian algorithm is a specialized method for solving the optimal matching problem in weighted bipartite graphs. It performs calculations with polynomial time complexity, making it particularly suitable for complex scenarios. In this context, the algorithm treats tasks as nodes in a bipartite graph and uses the distances between AGVs and tasks as weights to construct a matching matrix, ultimately determining the optimal allocation scheme. Therefore, we prefer to use the Hungarian algorithm to globally determine the optimal allocation, ensuring that each task is assigned to the most suitable AGV, minimizing the pretask path length, and prioritizing the execution of critical tasks. In practice, the Hungarian algorithm quickly computes the allocation of priority tasks based on the pre-calculated matrix $M_{f}$. Subsequently, using the endpoints of the priority tasks as the starting points for the AGVs, the PSO algorithm, as introduced earlier, is applied to determine the allocation of remaining tasks, minimizing the total no-load distance while ensuring a balanced task distribution.

The complete steps of the improved PSO algorithm, which accounts for both task balancing and task priority, are as follows:

Step 1: Initialize the order pool set Z, specify the starting point $S_{h}$ and endpoint $E_{h}$ for each task, and set the initial position $P_{w}$ for each $AGV_{w}$. Pre-calculate the distance matrix $M_{b}$ between tasks and the distance matrix $M_{s}$ from each AGV’s initial position to the starting points of the tasks.

Step 2: Initialize the priority task list $Z^{*}$ and construct the matrix $M_{f}$ from each AGV’s initial position to the starting points of the priority tasks.

Step 3: Assign priority tasks to the AGVs using either the greedy algorithm or the Hungarian algorithm.

Step 4: Initialize the positions $X_{i}$ and velocities $V_{i}$ of the particles based on the remaining tasks, and calculate the value of the optimization objective function $Q_{1}$.

Step 5: Initialize the algorithm control parameters, specifically the maximum number of iterations J and the population size N.

Step 6: Set the current iteration count j=1 and the current particle count i=1.

Step 7: Begin the j-th iteration:

Step 8: Optimize the i-th particle:

Step 9: Update the position and velocity of the particle according to Formulas (10) and (11), and perform constraint handling to ensure the feasibility of the solution.

Step 10: Calculate the optimization objective value $Q_{1}$ of the particle at the new position. If the new position is better than the previous $pbest_{i}$, update $pbest_{i}$ and the historical best value of particle i.

Step 11: Increment the current particle count i by 1. If i≤N, return to Step 8; Otherwise, all particles in this iteration have been optimized, and the best $Q_{1}$ value for the current iteration is recorded. If it is better than the current gbest, update gbest and the global best $Q_{1}$ value, then proceed to Step 12.

Step 12: Increment the current iteration count j by 1. If j≤J, return to Step 7; Otherwise, all iterations are complete and proceed to Step 13.

Step 13: Output the optimal task sequence that minimizes the no-load distance while fulfilling the task balancing and task priority requirements.

## Simulation test and analysis

To verify the effectiveness of the proposed multi-AGV multitask allocation optimization model and its solution algorithm, a testing environment based on the real factory production scenario in Fig 1 was established. The experimental results were then compared with those of the Multi-population Genetic Algorithm (MGA) [19] and the Market-based Bundle Task Allocation method (MBTA) [20]. The numbering of the grids follows a row-first manner, that is, starting from the first row, the first grid is numbered 1, and the 20 grids in each row are numbered sequentially to cover the entire factory area. From this, a grid connection graph can be constructed to store the entire factory terrain and facilitate the execution of subsequent algorithm. All algorithm codes were written in MATLAB R2021a and executed in an environment with an Intel Core i5-11320H 3.20 GHz (16.00 GB RAM) and Windows 11 operating system. The population size of MGA was set to 500, where four populations were created, each containing 125 individuals, while for our algorithm, the population size was set to 500. The maximum number of iterations was set to 100. The individual learning factor $c_{1}=2$, social learning factor $c_{2}=2$, inertia weight σ=0.9, grid side length r=1, and vehicle speed v=1.

### Algorithm effectiveness testing

To visually show the optimization effect of our algorithm for the basic problem model, 10 different tasks were generated in this test, as shown in Table 1.

**Table 1:** List of 10 simulated tasks.

Task number	Start point	End point	Task number	Start point	End point
1	40	293	6	388	260
2	94	292	7	378	271
3	220	385	8	344	19
4	176	109	9	251	400
5	377	186	10	24	145

**Fig 2:**
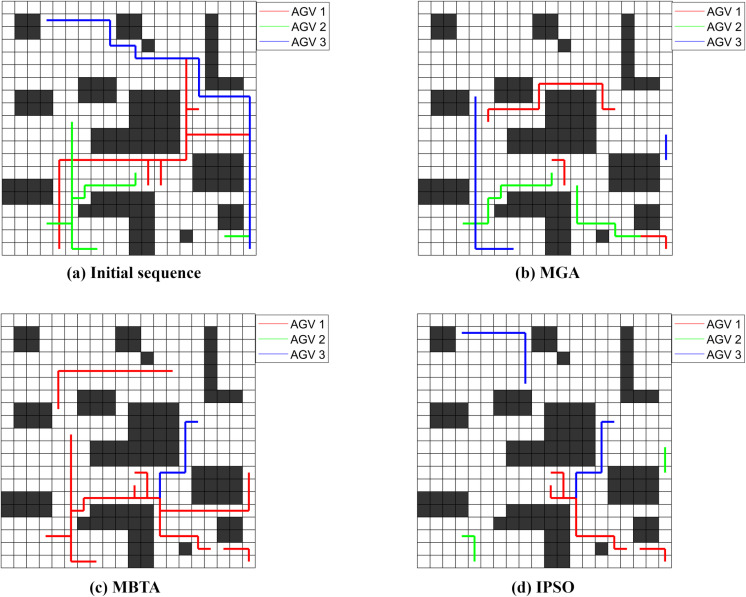
AGV inter-task paths based on the initial task sequence and three optimized task sequences.

We use three AGV vehicles to complete the tasks. The task execution order is based on the initial task sequence given in Table 1, the optimized task sequences calculated by MGA, MBTA, and our Improved PSO algorithm (IPSO), respectively. The driving paths between tasks were marked on the grid map to visually show the no-load distances between tasks of the three AGV vehicles, as shown in Fig 2.

Fig 2 shows that by optimizing the task execution order of the 10 tasks, the number of repeated driving paths is significantly reduced compared to the case based on the initial task sequence, and the AGV vehicle’s no-load distance between tasks is also greatly shortened. It can be calculated that the no-load distance between tasks of IPSO is 40, which is shorter than the distance 57 of MGA, the distance 73 of MBTA, and the distance 121 of the initial task sequence.

**Fig 3:**
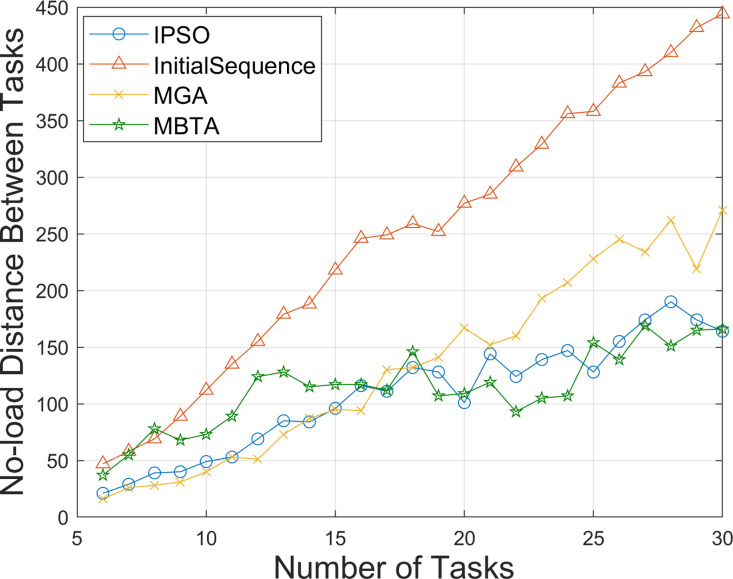
Comparison of the no-load distances between tasks under different numbers of tasks.

**Fig 4:**
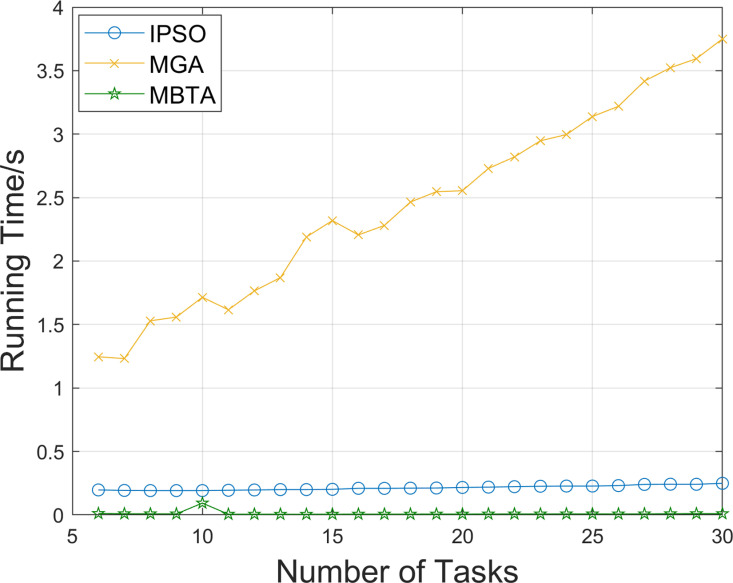
Comparison of algorithm running time under different numbers of tasks.

To further demonstrate the effectiveness of the improved PSO algorithm, we conducted comparative tests on different numbers of tasks using three AGV vehicles and recorded the changes in distance between tasks under the initial task sequence and three optimized task sequence. The results are shown in Fig 3, where the values presented are calculated based on averages of 10 runs for each test instance. It can be seen that in almost all test scales, the no-load distance between tasks under the optimized task sequence is significantly smaller than that under the initial task sequence, and this advantage becomes increasingly apparent as the number of tasks gradually increases from 6 to 30. In tests involving 6–17 tasks, the no-load distances achieved by IPSO and MGA are similar and both are lower than those of MBTA. After the 17th task, the no-load distances for IPSO and MBTA become similar, and both are lower than those for MGA. This demonstrates that IPSO performs well in reducing no-load distances across varying task numbers. The running times of the algorithms are shown in Fig 4. It can be observed that, under the same population size, IPSO runs much faster than MGA. As MBTA employs a non-iterative task assignment strategy, it runs the fastest. Moreover, as the number of tasks (i.e., problem size) increases, the running times of both IPSO and MBTA increase very slowly. These comparison results indicate that the improved PSO algorithm for the basic problem model is highly effective in optimizing the task sequences in the factory environment with multiple task requirements.

### Performance improvement on balanced task allocation

Since minimizing the no-load distance alone when calculating the optimal task sequence may result in a situation where a few AGVs perform most of the tasks while others remain idle, the improved PSO algorithm with balanced task allocation introduces a penalty term to mitigate such imbalances. To evaluate its performance, five AGVs were used to execute the tasks. The results of our method were then compared with those obtained from the MGA under the same optimization objective, as well as with those from the MBTA. The initial positions of the AGVs in the factory area of Fig 1 were set as follows: $P_{1}=1$, $P_{2}=5$, $P_{3}=10$, $P_{4}=15$, and $P_{5}=20$. The penalty coefficient was set to λ=10.

To reflect the workload and balance experienced by each AGV during task execution, we define the utilization rate of $AGV_{w}$ based on the no-load time $T_{idle}^{w}$, the task execution time $T_{load}^{w}$, and the posttask idle waiting time $T_{wait}^{w}$. A higher utilization rate indicates a more balanced task allocation and a more reasonable load distribution among the AGVs. The AGV utilization rate η is defined as follows:


$$\mathrm{\backslash [}\eta =\frac{1}{W}\sum_{w=1}^{W}\frac{T_{load}^{w}}{T_{idle}^{w}+T_{load}^{w}+T_{wait}^{w}}\mathrm{\backslash ]}$$ (18)

**Fig 5:**
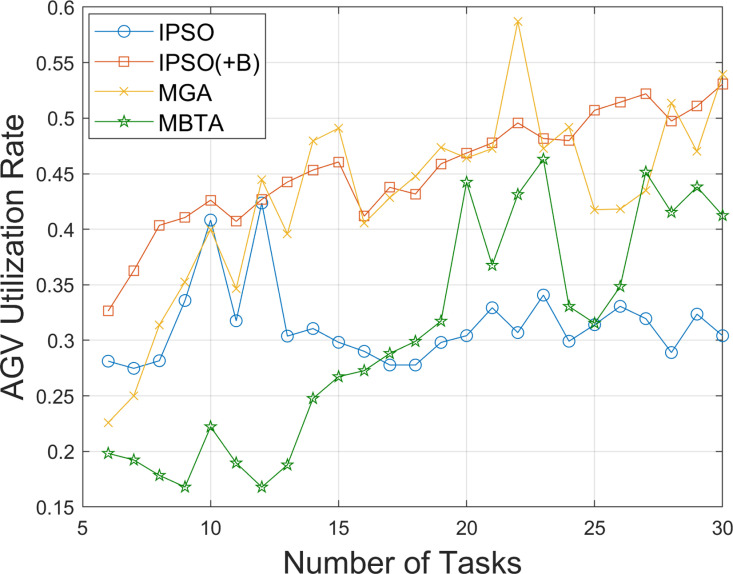
Comparison of AGV utilization rate under different numbers of tasks.

Fig 5 shows the change in AGV utilization as the number of tasks increases from 6 to 30, comparing the performance of IPSO, MGA, MBTA, and the Improved PSO algorithm with Balanced task allocation (IPSO(+B)). It can be observed that in all test instances, the average AGV utilization of the IPSO algorithm, which does not incorporate balanced task allocation, does not exceed 0.35. The MBTA method exhibits the lowest AGV utilization in tests with 6–16 tasks, with its utilization significantly improving only after the 16th task, reaching a mean value of 0.38. In contrast, IPSO(+B) and MGA achieve the highest AGV utilization in most test instances, with IPSO(+B) performing better overall. These results demonstrate the effectiveness of the improved balanced task allocation strategy in enhancing AGV utilization.

**Fig 6:**
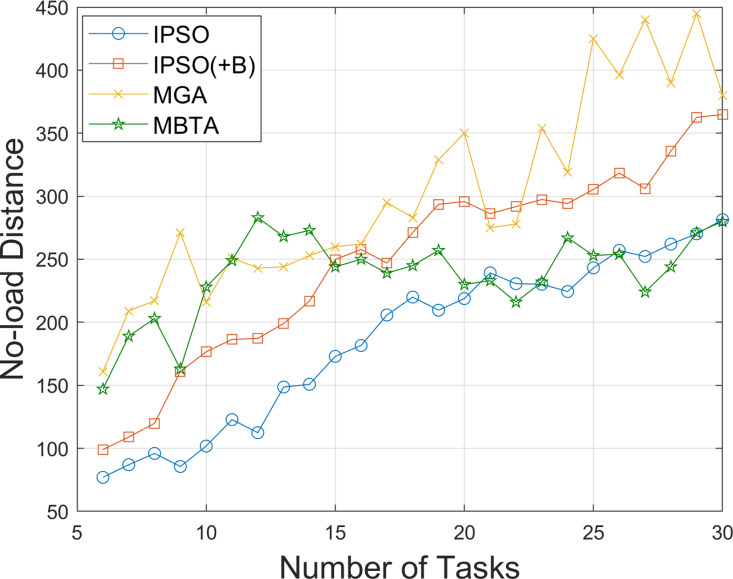
Comparison of total no-load distances under different numbers of tasks.

**Fig 7:**
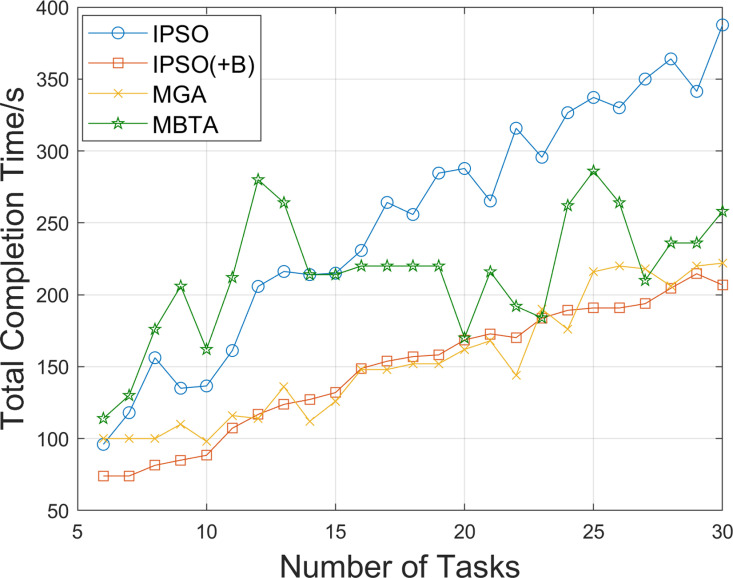
Comparison of total task completion times under different numbers of tasks.

As AGV utilization increases, the total time required to complete all tasks should decrease. To demonstrate this, we further compare the total no-load distance and total task completion time of the AGVs obtained using the above algorithms, as the number of tasks increases from 6 to 30. The results are shown in Figs 6 and 7. It can be observed that as the number of tasks increases, the total no-load distances of all algorithms gradually increase. Due to the adoption of a balanced task allocation strategy, the no-load distances for IPSO(+B) and MGA are higher than those for IPSO, with MGA exhibiting a larger increase. In tests involving 6–20 tasks, the no-load distances for MBTA are higher than those for IPSO, and after the 20th task, MBTA’s no-load distance becomes comparable to that of IPSO. However, the total task completion times for IPSO(+B) and MGA are significantly reduced. Overall, IPSO(+B) demonstrates excellent performance in both reducing the total no-load distance and minimizing the total task completion time.

To give an intuitive insight into the optimization effects, we take the instance of 30 tasks (as shown in Table 2) as an example to demonstrate the task allocation sequence, task completion time, and no-load distance of each AGV obtained using the above algorithms. The results are presented in Tables 3–6. It can be observed that IPSO, which does not incorporate balanced task allocation, exhibits the longest total task completion time but achieves the shortest no-load distance. MBTA, which also prioritizes minimizing the no-load distance, shows the second shortest no-load distance and the second longest total task completion time. In contrast, IPSO(+B) and MGA, both of which incorporate balanced task allocation, assign an average of six tasks to each AGV. This significantly improves AGV utilization, thereby reducing the total task completion time to 184 seconds and 232 seconds, respectively. Furthermore, compared to MGA, IPSO(+B) achieves both a shorter no-load distance and faster algorithm running time, highlighting the superior performance of the improved PSO algorithm with balanced task allocation proposed in this paper.

**Table 2:** List of 30 simulated tasks.

Task number	Start point	End point	Task number	Start point	End point
1	40	293	16	12	82
2	94	292	17	64	195
3	220	385	18	109	159
4	176	109	19	235	122
5	377	186	20	59	141
6	388	260	21	2	344
7	378	271	22	264	71
8	344	19	23	347	221
9	251	400	24	17	119
10	24	145	25	186	318
11	99	374	26	103	224
12	370	18	27	367	190
13	84	249	28	133	243
14	261	341	29	160	387
15	35	203	30	185	236

**Table 3:** Task sequences for each AGV obtained using IPSO.

Task queue	Time taken/s	No-Load distance
21-8-11-19-26-14-23-13-27-18-3-28-2	346	121
10	16	9
–	0	0
15-25-4	76	27
1-9-7-22-17-29-6-5-30-24-20-16-12	348	97

**Table 4:** Task sequences for each AGV obtained using IPSO(+B).

Task queue	Time taken/s	No-Load distance
21-23-9-1-2-20	184	74
26-30-3-27-28-12	162	50
15-25-7-19-18-8	178	63
4-13-14-17-29-10	182	91
24-11-5-22-16-6	160	56

**Table 5:** Task sequences for each AGV obtained using MGA.

Task queue	Time taken/s	No-Load distance
29-27-22-15-14-25	208	94
1-28-23-26-8-4	172	73
10-17-5-18-16-2	162	78
12-20-3-6-9-7	232	94
11-24-12-19-21-30	184	87

**Table 6:** Task sequences for each AGV obtained using MBTA.

Task queue	Time taken/s	No-Load distance
10-26-30-19-14-25-5-6-3-12	258	69
10-23-21-8-20	144	39
16-17-28	76	29
24-4-18-2-27-11-29	198	93
1-9-7-22-15	136	50

### Performance improvement on priority task allocation

Using the simulated task list in Table 2, five important tasks that need to be completed first are specified to evaluate the performance of the improved PSO algorithm with priority task allocation. The five priority task numbers are 3, 17, 18, 22, and 29. Under the premise of balanced task allocation, the effects of adopting the greedy algorithm and the Hungarian algorithm for assigning priority tasks to AGVs in the front end of the improved PSO algorithm are compared. The results are presented in Tables 7 and 8.

**Table 7:** Task sequences for each AGV when using greedy algorithm in the front end of IPSO(+B).

Task queue	Time taken/s	$L_{s}^{w}$	No-Load distance
17-9-1-23-12-16	147	6	69
3-6-19-26-22-11	169	25	82
22-2-5-25-28-14-13	198	19	89
18-20-30-27	151	11	56
29-8-24-4-18-15-7	182	7	77

**Table 8:** Task sequences for each AGV when using Hungarian algorithm in the front end of IPSO(+B).

Task queue	Time taken/s	$L_{s}^{w}$	No-Load distance
17-11-12-20-26-16	170	6	68
22-18-29-6-7-9	154	14	63
18-4-15-22-14	132	6	49
29-23-14-25-5-28	192	12	68
3-8-1-27-30-10-21	197	10	97

In both tables, the five priority tasks were assigned to each AGV first, with the remaining tasks arranged sequentially after the priority tasks. This ensures that priority tasks are processed first while simultaneously minimizing the total no-load distance. It can be observed that the two front-end algorithms generate completely different task execution sequences. Compared to the greedy algorithm, the task execution sequences calculated by the Hungarian algorithm result in a smaller pretask path length $L_{s}^{w}$ and total no-load distance, with the smaller pretask path length ensuring that priority tasks are started and completed earlier. As shown in Tables 7 and 8, the priority tasks numbered 3, 17, 18, 22, and 29 are indeed executed first, as compared to Table 4. Additionally, the AGV loads in Tables 7 and 8 are relatively balanced. This further demonstrates that the improved PSO algorithm, with its balanced and prioritized task allocation strategies, is highly effective and that the calculated task allocation sequences are reasonable.

## Conclusion

This study investigated the optimization problem of multitask allocation for multiple AGVs in a factory environment and established a mathematical model for the task allocation system. To address the varying no-load distances resulting from different task allocation combinations, optimizations based on allocation balance and task priority were incorporated into the PSO algorithm. By adding a penalty term to the optimization objective function, the algorithm ensures that no AGV remains idle due to the excessive pursuit of minimal no-load distances, thereby maintaining a balanced task allocation. Furthermore, by considering the initial positions of the AGVs and the sequence of prioritized tasks, the allocation methods preassign each priority task to an AGV, minimizing the total pretask path length. This ensures that priority tasks are started and completed as early as possible, thereby validating the rationality of the priority task allocation schemes. Simulation experiments validate the effectiveness of the improved PSO algorithm, along with the task balancing and task priority strategies proposed in this paper.

Although this paper presents preliminary research on the optimization problem of multi-AGV task allocation, several areas remain for further exploration. In practical applications, scenarios may arise where tasks have priority dependencies or undergo changes, such as when certain tasks must be executed after others. To address such situations, constraint terms could be introduced to extend the model. Additionally, the performance of the proposed algorithm can be further enhanced by adopting additional optimization strategies or combining it with other algorithms, such as genetic algorithm. As the number of AGVs and tasks increases significantly, methods like parallel computing should be explored to ensure that the algorithm can deliver more timely and accurate allocation solutions.

## Supporting information

S1 File Data and code used in this article. (ZIP)
